# Developing a congenital hyperinsulinism prioritized research agenda: a patient-driven international collaborative research network

**DOI:** 10.3389/fendo.2025.1549310

**Published:** 2025-05-02

**Authors:** Tai L. S. Pasquini, Indraneel Banerjee, Henrik Thybo Christesen, Louise S. Conwell, Antonia Dastamani, Diva D. De Leon, Sarah E. Flanagan, David Gillis, Jennifer M. Kalish, Katherine Lord, Mahlet Mesfin, Jennifer Schmitt, Senthil Senniappan, Charles A. Stanley, Paul S. Thornton, David Zangen, Julie Raskin

**Affiliations:** ^1^ Congenital Hyperinsulinism International, Glen Ridge, NJ, United States; ^2^ Department of Paediatric Endocrinology, Royal Manchester Children’s Hospital, Manchester, United Kingdom; ^3^ Steno Diabetes Centre, University of Southern Denmark, Department of Clinical Research, Hans Christian Andersen Children’s Hospital, Odense, Denmark; ^4^ Department of Endocrinology and Diabetes, Queensland Children’s Hospital and Children’s Health Queensland, Greater Brisbane Clinical School, Medical School, University of Queensland, Brisbane, QLD, Australia; ^5^ Department of Paediatric Endocrinology and Diabetes, Great Ormond Street Hospital for Children NHS Foundation Trust, London, United Kingdom; ^6^ Congenital Hyperinsulinism Center and Division of Endocrinology and Diabetes, Department of Pediatrics, Children’s Hospital of Philadelphia, Perelman School of Medicine at the University of Pennsylvania, Philadelphia, PA, United States; ^7^ Department of Clinical and Biomedical Science, University of Exeter Medical School, Exeter, United Kingdom; ^8^ Division of Pediatric Endocrinology, Hadassah Medical Center, Faculty of Medicine, Hebrew University of Jerusalem, Jerusalem, Israel; ^9^ Division of Human Genetics at the Children's Hospital of Philadelphia and the Departments of Pediatrics and Genetics at the Perelman School of Medicine at the University of Pennsylvania, Philadelphia, PA, United States; ^10^ Department of Paediatric Endocrinology, Alder Hey Children’s Hospital, Liverpool, United Kingdom; ^11^ Congenital Hyperinsulinism Center, Division of Endocrinology, Cook Children’s Medical Center, Fort Worth, TX, United States

**Keywords:** congenital hyperinsulinism, hypoglycemia, patient advocacy, rare diseases, prioritized research, collaborative research network

## Abstract

**Introduction:**

Congenital Hyperinsulinism (HI) is a rare disease that causes severe and recurrent hypoglycemia due to dysregulated insulin secretion. HI is the most frequent cause of severe, persistent hypoglycemia in newborns and children. Disease management is focused on preventing the neurological consequences associated with hypoglycemic brain injury; however, treatment is complex, often suboptimal, and places a large burden on families and individuals living with HI. Congenital Hyperinsulinism International (CHI) is an international patient organization that received a grant from the Chan Zuckerberg Initiative to establish the CHI Collaborative Research Network (CRN), a collaborative body to accelerate research for HI.

**Assessment process:**

Stakeholder groups relevant to HI, including individuals living with HI, families, researchers, clinicians, nurses, and industry partners, were identified to join the CRN and work together to create a prioritized research agenda (PRA) to systematically rank research priorities. CRN members worked across 7 workstream groups through a structured process to brainstorm gaps and corresponding solutions to formalize the HI PRA.

**Actionable recommendations:**

A total of 362 gaps were identified across research, infrastructure, knowledge, and funding. All groups identified the need for an HI Natural History Study; therefore, this item was identified as a priority that would automatically be placed on the finalized list. Other top gaps identified in the PRA addressed preventing brain damage and the need to increase awareness and understanding related to the role of early and effective diagnosis in preventing brain damage.

**Discussion:**

The formation of the CRN and the development of the PRA have already led to new collaborations, which are fundamental to progress. The PRA process allowed individuals to come to a consensus on the critical needs and to chart short- and long-term approaches to fill the gaps. CRN members continue to meet regularly in working groups focused on special projects to fill gaps identified as high priority by the PRA. Through this active and multidimensional alliance, the CRN is re-imagining the future for people living with HI by improving outcomes through more timely and accurate diagnosis, more effective and less burdensome treatments, more easily obtainable expert care, and better tools to manage HI.

## Introduction

Congenital Hyperinsulinism (HI) is a rare disease that causes severe and recurrent hypoketotic hypoglycemia due to dysregulated insulin secretion (1–4). HI is the most frequent cause of severe, persistent hypoglycemia in newborns and children (1, 2, 5, 6) and occurs in approximately 1 in 28,000 births in most countries (7, 8).

HI can be either transient or persistent. Transient HI is often due to acquired factors such as perinatal stress or gestational diabetes, but may still require treatment before the condition resolves (4, 9, 10). Roughly 50-60% of individuals with persistent forms of HI have an identified variant affecting one of over 30 different genes that control the insulin secretory pathway, with the most common cause being loss-of-function variants affecting the ATP-sensitive K^+^ channel (KATP-HI) (11, 12). In some individuals, HI is associated with a syndrome that affects multiple organ systems, including Beckwith-Wiedemann, Rubinstein Taybi, and Kabuki syndrome (13–15). Early diagnosis and effective treatment are critical to prevent brain damage and death that can be caused by prolonged hypoglycemia in both persistent and transient HI (2–4).

There is currently only one therapy option for HI with regulatory approval, diazoxide, which is only effective for a subset of patients (16–18). Off-label treatments include somatostatin analogs and glucagon (4). Many children also require frequent or continuous tube feedings with supplemental carbohydrates to maintain euglycemia. Pancreatic surgery is sometimes required for individuals with KATP-HI for both focal and diffuse forms of HI (4, 19). For individuals with focal KATP-HI, surgery can be curative if the lesion is completely excised. For individuals with diffuse KATP-HI who are unresponsive to medical therapy, surgical removal of 95-99% of the pancreas may be required (19). Individuals with diffuse disease may require multiple surgeries and can continue to require medications or frequent feeds to reduce the frequency of hypoglycemic events (19). Additionally, most individuals who have a subtotal pancreatectomy will go on to develop diabetes and pancreatic exocrine insufficiency (19–21). Even when on treatment, a subset of patients continues to experience frequent episodes of hypoglycemia (2, 22).

Preventing the neurological consequences associated with hypoglycemic brain injury is the number one concern of the HI community (4, 23). Many people with HI have additional physical, developmental, or psychological co-morbidities, some of which may be attributed to brain damage caused by prolonged hypoglycemia (22, 23). The most common include epilepsy and developmental delays. Cognitive deficits, speech problems, visual impairment, motor problems, learning challenges, and social-emotional problems are also frequently reported (3, 20, 22, 24).

Current screening practices are insufficient for the identification of newborns at risk of HI, whether transient or persistent, acquired or genetic (25). More than 40% of participants in the HI Global Registry (HIGR) did not have blood glucose screening before leaving the birth facility (22). HIGR is a registry that characterizes the experience of living with HI across a lifetime through a series of surveys completed by people living with HI, their caregivers, and their physician, and real-world data streams (22). All of these babies were later diagnosed with HI. Likely as a consequence of delayed diagnosis, up to half of the individuals with HI sustain hypoglycemia-induced brain damage (3, 20, 24, 26).

Feeding issues are also common for individuals living with HI and may be exacerbated by treatments and the need for frequent or continuous feeding. Young people growing up with HI often require multi-dimensional care, including regular psychosocial and family support. In many circumstances, including communities in the less affluent Global South, access to high-quality multidisciplinary care is often constrained by the availability of services or financial resources. Therefore, the treatment of HI is complex and often suboptimal, requiring considerable improvements in several aspects of the patient journey (2, 4).

There are limited studies available on the costs associated with HI care, but 59% of individuals in the HI Global Registry reported that their household income had been negatively impacted by the participant’s HI, with 38% reporting at least some difficulty paying for the costs associated with caring for the person with HI (27). In addition to direct costs, 39% of individuals in HIGR reported missing at least some work or school in the past year due to the participant’s HI (27). It is estimated that the direct costs of illness associated with HI patients in the United Kingdom covered by the National Health Service is £3.4 million annually, with the highest costs attributed to those in their first year of life (28).

### Prioritized research agenda

A prioritized research agenda (PRA) is a systematic ranking of research priorities agreed upon by stakeholders of a particular disease (29–31). The purpose of developing a PRA is to ensure research efforts align with the interests of members in a particular community, including individuals living with HI, caregivers, researchers, clinicians, and advocacy organizations. By defining the key areas of highest need, a PRA functions to efficiently guide research efforts toward the most relevant questions facing a disease community. A PRA aligns researchers and the community to areas of unmet clinical need and lays the groundwork for long-term grant funding and infrastructure to reduce barriers to ongoing collaboration and to establish larger multi-site research projects.

### Congenital hyperinsulinism international collaborative research network

Congenital Hyperinsulinism International (CHI) was founded in 2005 and is an international patient organization that is focused on awareness, research, and support, including helping families with HI worldwide (23). Since its founding, CHI has been assembling key stakeholders in the HI community for conferences to educate families, connect medical professionals, and seek better treatments and cures through global collaboration in a rare disease field.

In 2020, CHI received a grant from the Chan Zuckerberg Initiative for the Rare as One Network to establish the CHI Collaborative Research Network (CRN). The focus of this initiative was to provide patient organizations with the capacity building, funding, and tools to launch and lead a collaborative research network (32). There is growing evidence that patient-centered approaches increase the alignment between clinicians and patients and are more likely to address real-world needs and concerns (33–35). While there had been HI research collaborations between HI expert scientists and clinicians in the past, the CHI CRN was the first collaborative research body to not only include people affected by the condition but also have them lead it.

The grant structure led to the identification of a Lead Clinician (Paul Thornton, Cook Children’s Medical Center), Lead Researcher (Diva D. De León-Crutchlow, Children’s Hospital of Philadelphia), and patient organization lead (Julie Raskin, CHI Chief Executive Officer). These individuals, along with two other CHI employees (Tai Pasquini, Chief Research Officer, and Jennifer Schmitt, Chief Operating Officer), became the CRN core team. For the first year of the CRN, the core team established two main goals, 1) develop the infrastructure and long-term viability of the CRN and 2) formalize a PRA for HI.

The CRN’s Mission is to “Create a hyperinsulinism collaborative research network that puts patients at the center of a strategy that leads to faster and more accurate diagnosis, drives new evidence-based treatments and cures, standardizes clinical guidelines, and facilitates increased and improved access.”

The CRN established a set of cornerstones to guide the principles of the program, including a commitment to:

- Supporting collaboration across the globe;
- Elevating the patient voice and ensuring the patient perspective is central to our work;
- Guaranteeing access to information, medical specialists, and treatments regardless of income and geography;
- Engaging new researchers and ideas to find innovative concepts and foster additional leaders in the HI network; and
- Addressing diversity, equity, and inclusion in our work and our community.

The CRN was launched to the HI community in December 2020 at a virtual convening with 200 participants. At this initial convening, the concept of the CRN was introduced, along with a plan of work to develop a prioritized research agenda for HI through visioning and brainstorming.

## Assessment process to evaluate research gaps and activities

**Figure 1:**
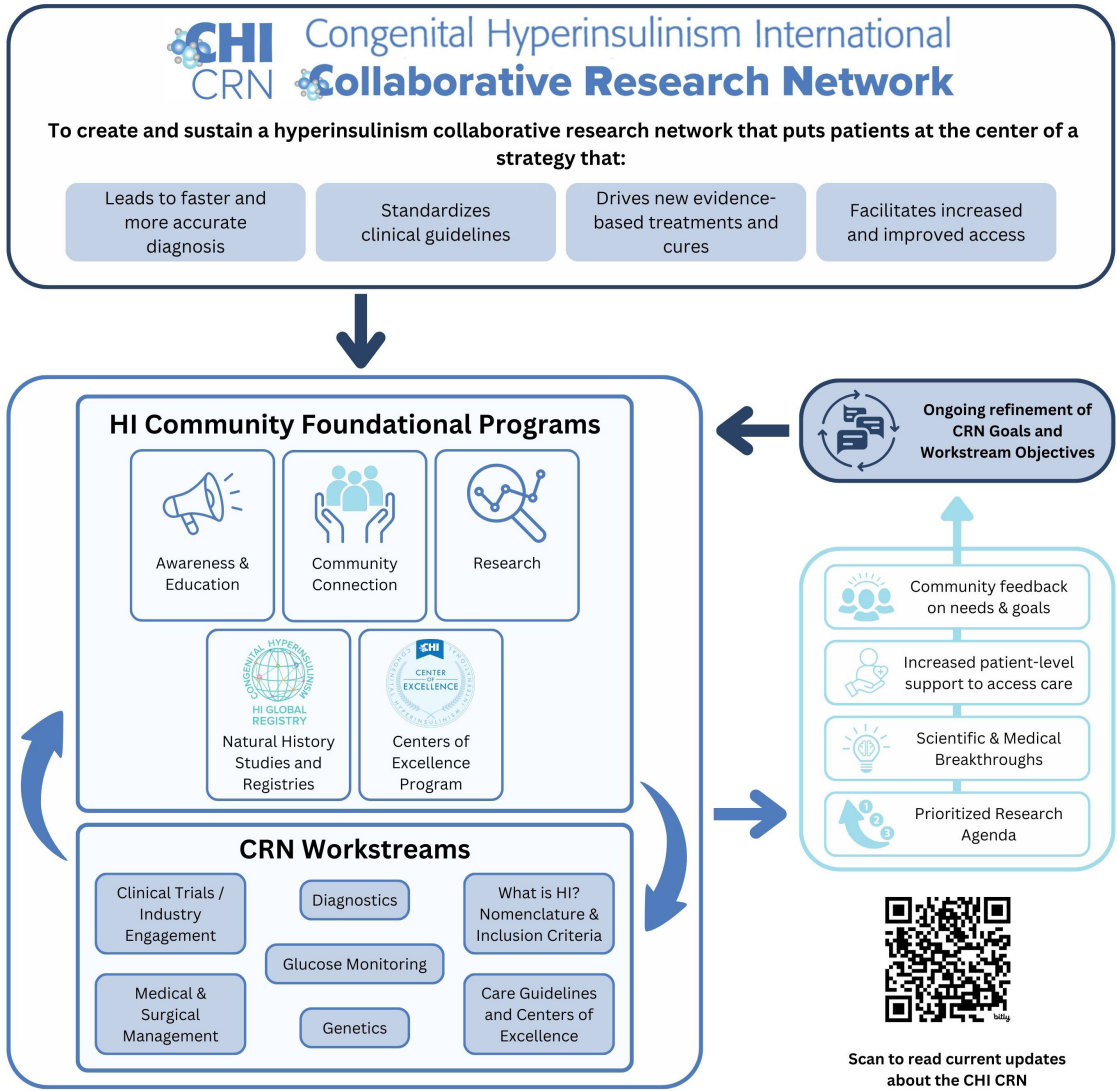
Collaborative research network approach.

Stakeholder groups relevant to HI, including individuals living with HI, families, researchers, clinicians, nurses, and industry partners, were identified, and in March 2021, 57 people were invited by the core team to join one of the 7 CRN workstream groups as members (**Figure 1**). Health professional and scientific researcher members were identified based on contributions to manuscripts, ongoing collaborations, and participation in HI family conferences. Each group had a patient or caregiver representative identified based on their role as an international patient group leader or their diverse experiences and skill sets applied to the promotion of the HI global community. Each group had a chair or co-chair to serve as the leader who worked closely with the core team to guide each group through a year-long consensus process to formalize the HI PRA.

**Figure 2:**
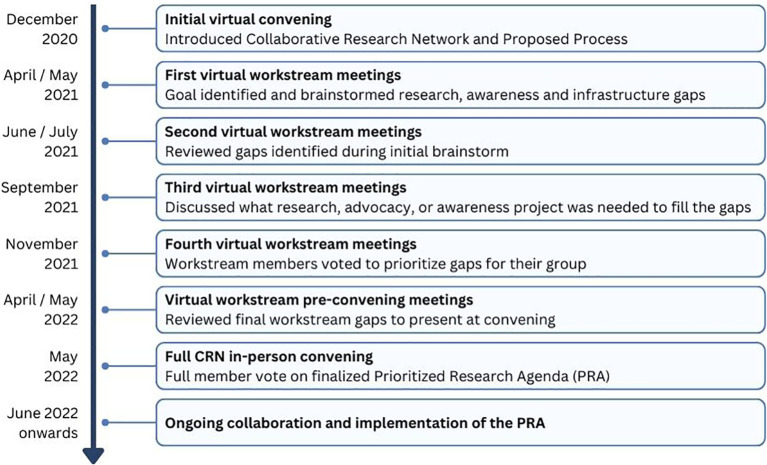
Collaborative research network meeting process.

During the first workstream meetings, each group formalized its goal or vision and discussed the importance of its topic within the context of HI (**Figure 2**). Then, individuals were asked to brainstorm what they saw as the “significant gaps” for their topic. This included gaps in any of the following categories: research gaps or needs, infrastructure/pooled research resources/collaboration, knowledge/expertise, or dissemination/funding.

During the second round of workstream meetings, individuals reviewed the group brainstorming document, added any missing gaps, and consolidated the ideas into categories. The CHI team edited the gaps for clarity and consistency across workstreams.

The third set of workstream meetings focused on describing the elements that were needed to fill the gaps. These could be research components or actions (specific research studies) or tools (software or infrastructure). The groups also discussed current initiatives in progress, estimated expenses, and timeframes needed to fill the gap.

During the fourth meeting, the goal was to prioritize gap statements. When a common gap item appeared in multiple workstream groups, the item was assigned to one group for prioritization within the final PRA. During the meeting there was a live poll conducted for each of the gaps identified by the group. Members were asked to consider:

- Whether the proposed research or action centered on the mission of the CRN as a whole and on the mission of the workstream
- The likelihood that the proposed research or action will improve diagnostic or clinical practice or clinical decision-making
- Whether the project would yield a good return on investment in the short and long term
- Applicability of the proposed project to other workstream projects

Each person could respond on a 3-point scale with Disagree (0 points), Agree (1 point), or Strongly Agree (2 points). An overall score was generated for each item. During the final workstream meetings, CRN members reviewed the voting results and made final recommendations on items to share with the full CRN for consideration for the final PRA. Each workstream group chose between 4 and 5 gap items to share with the full CRN group. The workstream groups worked on combining items and updating language to reflect the workstream’s discussions and intent.

In May 2022, the full CRN was assembled for the first time in person in Lisbon, Portugal. During the meeting, each group presented the top gap items identified within their workstream, followed by CRN member voting to rank each item to establish the final list.

## Actionable recommendations

A total of 362 gap items were identified across research, infrastructure, knowledge, and funding. This section will review the rationale and major goal of each workstream group, and the priorities identified first by the workstream groups and then the results that became the final PRA.

### Care guidelines and centers of excellence

#### Co-chairs: Henrik Christesen and Louise Conwell

This group was formed because access to quality care is critical for patients regardless of geography. HI management is complex, and collaboration across expert centers to share data and establish quality metrics will improve knowledge and patient outcomes. Finally, understanding the use of existing care guidelines for HI and areas for development ensures the continuous evolution of quality clinical care.

The vision of the Care Guidelines and Centers of Excellence (COEs) workstream is a better future for those with HI through improved care guidelines, centers of excellence, and collaboration to improve the quality of life and outcomes for individuals living with HI and their families.

The Care Guidelines and Centers of Excellence group identified a total of 57 gaps. The top research gaps identified were (**Table 1**):

- Continually evolving global care guidelines
- A structure and network of HI centers
- Data collection to inform clinical care
- Inventory of global access
- Data analysis to inform clinical care and quality metrics

**Table 1:** CHI CRN care guidelines and centers of excellence top 5 prioritized research agenda gaps.

Gap	Overview of activities needed to fill the gap
Continually evolving global care guidelines	Collaborate with specialists across medical disciplines to identify current standards of care, identify gaps, and publish clinical care guidelines for all phases of HI, including pre-diagnosis. Ensure that the guidelines include the family perspective, are accessible to the lay reader, and are available in different languages. Generate a plan for continuing review of the guidelines to ensure that they appropriately reflect changes to standards of care as they evolve over time.
A structure and network of HI centers	Create a network of centers of excellence (COE) that encourages partnership with smaller centers, training opportunities, and a leveled approach for centers in different contexts (health-resourcing, socio-economic, etc.). Ensure caregiver/patient preferences and opinions are integrated into various care components. Ensure all care guidelines adapt to diverse environments.
Data collection to inform clinical care	Utilize the COE structure for data collection. Increase partnerships with HI Global Registry (HIGR), HI Centers, and other relevant registries, including expanding participation to centers/countries not currently participating. Work with global endocrinology groups to engage more pediatric endocrinologists internationally. Agree upon HI common data elements, definitions, and outcome measures to ensure optimal patient outcomes.
Inventory of global access	Inventory access to medications (HI medications and HI-related medications), treatment, surgery, devices (CGM, glucometer), supplies, F-DOPA, PET/CT, MRI, and genetics (functional studies) globally. Strengthen data collection at the national/local level and then collect it centrally.
Data analysis to inform clinical care and quality metrics	Assess the efficacy of different healthcare delivery models (e.g., telemedicine, fasting studies, and dosing). Identify what is considered a “good” outcome, including increasing understanding of what happened before a patient went to a center to contextualize outcomes. Define, standardize, and compare metrics on process, structure, quality of care, and outcomes for care guidelines and COEs. Assess care guidelines uptake and implementation.

### Glucose monitoring

#### Co-chairs: Antonia Dastamani and David Zangen

Glucose monitoring is a daily part of living with HI for patients and families. Devices are critical tools for daily management and improvements to their design and accessibility will support people living with HI (36–39). Glucose monitoring is also critical for HI research and understanding key aspects of meaningful glycemic thresholds and patient outcomes. This group was formed to examine all aspects of current and future glucose monitoring techniques in clinical and at-home care.

The glucose monitoring workstream envisions a better future for those with HI through improved glucose monitoring, which will improve patients’ quality of life, diagnostics, and outcomes.

The glucose monitoring group identified a total of 42 gaps. The top research gaps identified were (**Table 2**):

- Continuous Glucose Monitoring (CGM) use
- Understanding of the impact on patient experience using home devices to monitor blood glucose
- Miniaturized electroencephalogram (EEG) and blood glucose monitoring study
- Device technology for HI patients
- Access to CGM technology for all patients

**Table 2:** CHI CRN glucose monitoring top 5 prioritized research agenda gaps.

Gap	Overview of activities needed to fill the gap
Continuous Glucose Monitoring (CGM) use	Utilize clinical data as part of research studies, including longitudinal CGM data and NICU studies, compared with neurologic outcomes amongst different sub-groups of HI patient type and accuracy at various ages.
Understanding of the impact on patient experience using home devices to monitor blood glucose	Determine the impact of using CGM at home (i.e., anxiety, independence). Conduct patient preference surveys and patient use surveys.
Miniaturized electroencephalogram (EEG) and blood glucose monitoring study	Complete miniaturized electroencephalogram (EEG) and blood glucose study. Identify partnerships for studies with EEG device companies.
Device technology for HI patients	Improve technology for HI care with companies, Research and Development teams, and regulatory agencies for device indications. Increase accuracy in lower ranges.
Access to CGM technology for all patients	Streamline device approval pathways for HI. Write shared letters of medical necessity. Create advocacy plans or strategies for companies, payers, and regulatory agencies.

### Genetics

#### Co-chairs: Sarah Flanagan and Jennifer Kalish

The genetics group was formed because understanding genetics is critical for diagnosis, treatment decisions, and prognosis. Additionally, identifying new genetic variants is key to further understanding the underlying cause of HI (1, 40). Currently, only about half of those who have had targeted genetic testing for HI have received a genetic diagnosis (41, 42). Future research is critical to unlocking new diagnostics and treatments and providing crucial information for family planning.

The vision set forth by the genetics workgroup is a better future for those with HI through an improved understanding of genetics for a better quality of life and outcomes for patients.

The genetics group identified a total of 46 gaps. The top research gaps identified by the genetics group included (**Table 3**):

- Access to genetic testing
- Undiscovered genetic variants
- Relationship between genetics and treatment
- Variants in known genes
- Relationship between HI and genetic syndromes

**Table 3:** CHI CRN genetics top 5 prioritized research agenda gaps.

Gap	Overview of activities needed to fill the gap
Access to genetic testing	Understand the insurance coverage of genetics. Determine what genetic testing is needed, develop a standard of care, and improve access to comprehensive HI genetics diagnostics and research, including cascade testing for extended family members and urgent panel testing. Educate healthcare professionals who are ordering, interpreting, and sharing test results.
Undiscovered genetic variants	Investigate epigenetic signatures that are specific to HI and study the regulation of gene expression using RNA sequencing analysis and animal models. Use clinical characterizations to determine the likelihood of a monogenic diagnosis, including syndromes associated with HI. Use clinical correlation and education to match genomic to clinical data for studies. Understand how people respond to drugs to uncover which pathways are most likely disrupted by genetic variants.
Relationship between genetics and treatment	Identify novel drug targets and group treatments according to the disrupted biological pathways. Prepare for “personalized medicine” for specific disease-causing variants, including using induced pluripotent stem cells (iPSCs), animal models, gene therapy, and cell models to examine disease mechanisms and drug response. Research genetic modifiers to better understand clinical heterogeneity within genetic/syndromic forms of HI. Share existing knowledge of how a specific genotype impacts on drug response for physician use.
Variants in known genes	Identify fetal genotype using cell-free DNA in a maternal blood sample. Improve interpretation of new variants, including clinical correlation and functional studies. Improve pancreatic RNA/DNA detection in peripheral blood when a variant is only present within the pancreas. Re-test historic cases. Connect genetics to clinical data and HIGR data.
Relationship between HI and genetic syndromes	Utilize shared platforms for collecting genetic information. Understand the molecular mechanisms of syndromic causes of HI. Collect clinical information for individuals with genetic syndromes. Monitor HI patients to understand the potential need to diagnose other syndromes. Raise awareness among physicians of other related syndromes.

### Diagnostics

#### Chair: Katherine Lord

An accurate and timely diagnosis is critical to reduce the impact of prolonged hypoglycemia, including its impact on the brain. More precise tools, diagnostic pathways, and assessment protocols to aid in diagnosis can be life-altering (4, 25, 43, 44). Diagnosis was identified as a critical area due to its foundational importance for long-term outcomes for individuals with HI.

The vision set forth by the diagnostics workgroup is a better future for those with HI through improved diagnostics for a better quality of life and outcomes for patients. ​

The diagnostics group identified 55 individual gaps. The top research gaps presented by this group included (**Table 4**):

- Glucose as a vital sign
- Global HI mutation database
- A biomarker for hyperinsulinism
- Identification and adoption of newborn screening approach
- Shorter protocols for (18) F-FDOPA (6-[18F]-L-fluoro-L-3, 4-dihydroxyphenylalanine) (18FDOPA) PET scanning

**Table 4:** CHI CRN diagnostics top 5 prioritized research agenda gaps.

Gap	Overview of activities needed to fill the gap
Glucose as a vital sign	Increase awareness and timely diagnosis of HI, including through advocacy and awareness activities to encourage all healthcare providers to take low blood glucose seriously. Research glucose thresholds for diagnosis and prospective cohort studies to understand long-term outcomes based on early neonatal glucose levels. Identify barriers to healthcare providers recognizing hypoglycemia and risks associated with low glucose levels.
Global HI mutation database	Partner for a global HI mutation database that includes stored whole-exome sequencing (WES) data for unfound genetic mutations.
A biomarker for hyperinsulinism	Identify a biomarker for HI. Work with metabolomic experts for biomarkers and to compare biological samples from babies with and without HI to facilitate early diagnosis through prenatal or post-birth neonatal testing.
Identification and adoption of newborn screening approach	Understand the use of screening options during universal screening/blood spot tests. Define the thresholds and actions that need to be taken. Develop a test that could be included in the newborn screening program.
Shorter protocols for FDOPA PET scanning	Determine if necessary diagnostic information can be obtained from shorter-duration scans.

### Medical and surgical management

#### Co-chairs: Indi Banerjee and David Gillis

The purpose of establishing the medical and surgical management workgroup was to understand both current and future treatments and cures for HI. Managing HI is dependent on robust clinical information and a thorough understanding of its natural history. New approaches to medical and surgical treatments rely on consistent pooled data and tools to compare treatment approaches. Currently, effective treatments are not available for all types of HI, and even for patients who may have a treatment option, it may be inadequate or cause side effects (2). Even amongst the leading care centers, there are differences in approaches that are important to understand to achieve the best outcomes.

The medical and surgical management workgroup envisions a better future for those with HI through new and better medical and surgical treatments or cures.

The medical and surgical management group identified 77 individual gaps. This group also had the highest level of overlap with other groups and “endorsed” items that other groups presented at the convening. The final research gaps presented by this group included (**Table 5**):

- Knowledge of the cause of neurological damage
- Implementation of personalized medicine
- Drug design and repurposing
- New and more efficient surgical techniques
- A non-surgical method to target focal lesions

**Table 5:** CHI CRN medical and surgical management top 5 prioritized research agenda gaps.

Gap	Overview of activities needed to fill the gap
Knowledge of the cause of neurological damage	Understand how, when, and why brain damage occurs to understand protective factors and correlate glucose change over time. Conduct MRI studies at diagnosis and at regular intervals.
Implementation of personalized medicine	Conduct basic science and functional studies in patient tissues. Pursue individualized glucose handling and tolerance. Advanced imaging techniques for beta cell function and beta cell mass. Create glucose-sensing glucagon/insulin administration devices. Develop gene therapies for functioning beta cells.
Drug design and repurposing	Find efficient and well-tolerated treatments for all patients. Standardize the outcome measures for investigating new therapies. Design long-term outcome studies to understand the different approaches for equivalent therapies. Conduct head-to-head clinical trials to compare current off-label treatments. Design registries for post-marketing surveillance.
New and more efficient surgical techniques	Initiate studies to identify tissue-sparing pancreatic surgery. Identify intraoperative techniques to replace FDOPA. Harvest islet cells for genetic engineering. Find ways to encourage the regeneration of pancreatic cells.
A non-surgical method to target focal lesions	Identify approaches similar to thyroid and cancer radioisotope treatment to target focal HI. Link toxic treatment to specific molecules in hyperfunctioning beta cells. Understand why beta cells are abnormal in focal HI. Develop methods to target the destruction of local tissue.

### What is HI? nomenclature and inclusion criteria

#### Co-chairs: Charles Stanley and Senthil Senniappan

The purpose of establishing the What is HI? workgroup was to bring clarity to how the HI patient community is defined and characterized. Currently, there is no universal agreement on the name of the condition, its abbreviation, and subtypes. Important disease descriptors exist that capture some of the heterogeneity, but the lack of standardization is a problem for raising awareness of the condition in the medical and patient communities and for data collection for research purposes. Nomenclature that is universally adopted and an awareness campaign to disseminate it is necessary. The word congenital in the current name, “congenital hyperinsulinism,” is problematic because the patient community includes people with acquired hyperinsulinism.

Patients and their families who have been affected by HI but who are no longer treated for hypoglycemia may not consider themselves part of the HI community. It is important that all who have ever experienced HI consider themselves having a place in the HI community. This is important because there are often secondary issues to HI, and it is important to support these individuals throughout the lifecycle.

The vision set forth by the What is HI? Nomenclature and Inclusion Criteria workstream is to develop a plan to bring synergy to the way the patients, physicians, and medical industry decision makers describe the disease, to better define who is counted in the “HI patient community,” to agree upon a set of terms that define the condition and its subtypes, and to educate all appropriate stakeholders.

The group identified 27 individual gaps. The top research gaps identified by this workstream group included (**Table 6**):

- Develop a universal nomenclature for all forms of HI
- Dissemination of educational materials to different stakeholder groups (e.g., HI families, medical professionals, medical plans or payors, the public)
- Develop international classification of disease (ICD) and other relevant codes for HI
- Use of nomenclature for diagnostics: Better names for diagnostic testing to determine the cause of hypoglycemia and type of HI
- Nomenclature for HI drugs and devices to facilitate access

**Table 6:** CHI CRN What is HI? nomenclature and inclusion criteria top 5 prioritized research agenda gaps.

Gap	Overview of activities needed to fill the gap
Develop a universal nomenclature for all forms of HI	Engage world endocrine societies, neonatal experts, adult endocrinologists, and others to discuss consistent nomenclature and to understand cross-discipline-specific needs. Ensure that nomenclature resonates across languages and countries. Decide who gets included as having/had HI.
Dissemination of educational materials to different stakeholder groups (HI families, medical professionals, medical plans, the public, etc.)	Create more lay-language educational resources. Strengthen partnerships across countries to share information in multiple languages/countries/cultures. Grow an outreach and engagement campaign to anyone who has or has ever had hypoglycemia due to HI. Collaborate with neonatologists and pediatricians caring for babies worldwide to enhance knowledge.
Develop ICD and other relevant codes for HI	Engage coding experts to understand best practices for coverage in multiple international coding contexts. Lobby for and adopt appropriate codes that reflect HI nomenclature.
Use of nomenclature for diagnostics: Better names for diagnostic testing to determine the cause of hypoglycemia and type of HI	Develop a proposal for the nomenclature of HI diagnostics, including research into how to “rename” diagnostic procedures. Conduct advocacy efforts to disseminate and aid in the adoption of nomenclature changes.
Nomenclature for HI drugs and devices to facilitate access	Document the need for tools and supplies approved for other conditions that are used for HI patients. Utilize drug and device development. Increase insurance approval through letters of medical necessity and advocacy payers.

### Clinical trials/industry engagement

#### Co-chairs: Julie Raskin and Tai Pasquini

One of the unique features of the CHI CRN is the inclusion of biotechnology companies actively working on the development of HI treatments. The Clinical Trials/Industry Engagement group was established to facilitate conversations about the non-competitive shared goals and initiatives that would benefit the entire community. Clinical trials are crucial in the development of safe and effective new treatments and cures for HI patients. This group was led by the patient organization to maximize collaboration with all stakeholders and to find innovative ways to accelerate research for new treatments and cures.

The vision set forth by the clinical trials and industry engagement workstream is a space where patient and industry leaders and academic researchers and clinicians can come together to consider collaborations and approaches to enable progress in clinical research for today’s projects and tomorrow’s innovations.

In addition to the areas identified by the other groups, this group also categorized gaps related to the design and operations of clinical trials and the regulatory process. This group identified 58 individual gaps. The final research gaps the group presented were (**Table 7**):

- An expert group to develop novel clinical trials and outcome measures for regulatory approval
- Understanding of the burden of disease
- Meaningful glycemic endpoints
- Expand access and accelerate enrollment in clinical trials

**Table 7:** CHI CRN clinical trials/industry engagement top 4 prioritized research agenda gaps.

Gap	Overview of activities needed to fill the gap
An expert group to develop novel clinical trials and outcome measures for regulatory approval	Bring together experts to develop and propose a strategy to regulatory bodies for endpoints, outcomes, and clinical trial design. Align biotech companies and PI institutions to accelerate clinical trials. Conduct a survey for centers not participating in trials to understand barriers to being trial sites. Design a single-arm clinical trial study for treatment approval to reduce the risk of exposure to a placebo.
Understanding of the burden of disease	Measure the cost of uncontrolled disease. Understand how hypoglycemia and fear of hypoglycemia are connected to clinical outcomes, quality of life, and burden of disease for the person with HI and family. Share how the adverse effects impact life and burden of disease. Understand direct and indirect costs, including those associated with medication impact.
Meaningful glycemic endpoints	Understand time in range and derivation, including across tools and devices. Determine what percentage of time in range is clinically significant. Conduct a study to determine if there are incremental improvements in neurologic outcomes.
Expand access and accelerate enrollment in clinical trials	Increase educational awareness campaigns with other patient advocacy and rare disease organizations. Identify better ways to reach potential trial participants to optimize recruitment and decrease the burden for participants. Work with umbrella organizations and other advocacy groups to reduce barriers to cross-country participation.

### Natural history studies

Prior to voting, all groups identified the need for an HI Natural History Study to fill a key gap. Therefore, this item was identified as a top priority across all groups that would automatically be placed on the finalized list. Necessary actions included building a robust registry that collects patient-reported, physician-reported, and real-world data to provide a foundation for an HI natural history study.

HIGR and institutional registries were identified as the foundation for these efforts. HIGR is comprised of 13 patient/caregiver surveys and one physician survey and real-world data streams. Specific aims for the gap, “expansion of a robust natural history study” included understanding how hypoglycemia is connected to clinical outcomes, quality of life, and burden of disease, and the glycemic variability across patient phenotypes, ages, genetic subtypes, and any natural resolution by age.

### Final prioritized research agenda

The top gaps identified in the PRA all addressed brain damage and the need to increase awareness and understanding related to the role of early and effective diagnosis in preventing brain damage (**Table 8**). Critical work and next steps were identified from every workstream group.

**Table 8:** Final Congenital Hyperinsulinism International Collaborative Research Network prioritized research agenda.

Rank	Workstream	Gap/Need
1	All	HI Natural History Study
2	Diagnostics	Identification and adoption of newborn screening approach
3	Diagnostics	Glucose as a vital sign
4	Medical and Surgical Treatments	Knowledge of the cause of neurological damage
5	Care Guidelines & Centers of Excellence	Continually evolving global care guidelines
6	Clinical Trials & Industry Engagement	An expert group to develop novel clinical trials and outcome measures for regulatory approval
7	Genetics	Access to genetic testing
8	Diagnostics	A biomarker for hyperinsulinism
9	Clinical Trials & Industry Engagement	Meaningful glycemic endpoints
10	Glucose Monitoring	Device technology for HI patients
11	Medical and Surgical Treatments	Implementation of personalized medicine
12	Clinical Trials & Industry Engagement	Expand access and accelerate enrollment in clinical trials
13	Genetics	Relationship between genetics and treatment
14	What is HI: Nomenclature and Inclusion	Dissemination of educational materials to different stakeholder groups
15	What is HI: Nomenclature and Inclusion	Universal nomenclature for all forms of HI
16	Clinical Trials & Industry Engagement	Understanding of the burden of disease
17	Glucose Monitoring	Understanding of the impact on patient experience using home devices to monitor blood glucose
18	Glucose Monitoring	CGM use study
19	Care Guidelines & Centers of Excellence	Inventory of global access
20	Glucose Monitoring	Access to CGM technology for all patients
21	Care Guidelines & Centers of Excellence	A structure and network of HI centers
22	Diagnostics	Global CHI mutation database
23	Genetics	Undiscovered genetic variants
24	Medical and Surgical Treatments	Drug design and repurposing
25	What is HI: Nomenclature and Inclusion	ICD and other relevant codes for HI
26	Care Guidelines & Centers of Excellence	Data collection to inform clinical care
27	Care Guidelines & Centers of Excellence	Data analysis to inform clinical care and quality metrics
28	Genetics	Variants in known genes
29	Genetics	Relationship between HI and genetic syndromes
30	What is HI: Nomenclature and Inclusion	Nomenclature for diagnostics
31	What is HI: Nomenclature and Inclusion	Nomenclature for HI drugs and devices to facilitate access
32	Diagnostics	Improved radio imaging for hyperinsulinism research
33	Glucose Monitoring	Miniaturized EEG and blood glucose monitoring study
34	Medical and Surgical Treatments	New and more efficient surgical techniques
35	Medical and Surgical Treatments	A non-surgical method to target focal lesions​

## Discussion

While there have been significant advances in the understanding and treatment of HI over the last few decades, including a surgical cure for focal hyperinsulinism, galvanizing the full community of stakeholders to go through a process of prioritization was needed. Due to the continued significant unmet need that still exists for people living with congenital hyperinsulinism, research prioritization for better screening practices, treatments, cures, and improved quality of life was essential. The CHI CRN organized clinical, research, and scientific HI experts to work together on this prioritization and has importantly added patient and caregiver perspectives to the very core of the project’s leadership.

The PRA represents a very important step forward for the global HI community. The PRA process allowed individuals to come to a consensus on the critical needs and to chart short- and long-term approaches to fill the gaps. The formation of the CRN and the development of the PRA has already led to new collaborations, which are fundamental to progress. CRN members continue to meet regularly in working groups focused on special projects to fill gaps identified as high priority by the PRA.

One of the PRA process’s greatest strengths was the involvement of different stakeholder perspectives, including medical researchers and patient and caregiver experts. As the process began during the global pandemic, collaborating online was both timely and easier than it would have been if the CRN had launched previously. Members had become acquainted with virtual, online meeting platforms. Members were more available for meetings than at any other time previously, due to Covid 19 lockdowns. Additionally, the project benefitted from a historic baseline of collaboration across CHI family conferences and scientific partnerships among many of the member clinician-scientists.

One limitation of the initial CRN membership was that some valuable perspectives were absent from the original composition of the CRN, such as there were no neonatologists and there was just one adult living with HI. Although 16 countries were represented, there was also a desire to have additional members from other geographic regions. Since the PRA process, the CRN membership has expanded to include these additional perspectives including neonatologists, more adults living with HI, and individuals from additional countries. Additionally, we have begun more collaborations with individuals from outside of the HI community to help us learn from the best practices of related fields. The meeting process was also rigorous and required a large time commitment from individuals, which was often difficult to accommodate across time zones and professional responsibilities.

The most pressing issues identified through the PRA, including a natural history study, universal newborn screening approaches, and evolving care guidelines, require multi-center long-term studies an approach further cultivated by the establishment of the CRN. Of the 35 issues brought to the in-person convening, 63% either have an official CRN project, are being addressed by a member of the CRN, or discussions have begun to launch an initiative that addresses some of the components.

Since the launch of the CRN in 2020, the CRN members have published a total of 226 articles on congenital hyperinsulinism in the areas of genetics, treatment and management, monitoring and technology, screening and diagnostics, guidelines and education, patient experience and quality of life, and policy and access. The CRN is also working collaboratively on global advocacy and dissemination of information in addition to continuing to focus on collaborative research initiatives including updating educational materials, including the Wikipedia page for HI (https://en.wikipedia.org/wiki/Congenital_hyperinsulinism), to provide reliable and up-to-date information.

The development of the PRA reinforced the need for consistent and sustained collaboration, which has continued to grow through the CRN, including an HI nurse working group and an increased focus in the community on newborn screening and global access. Members of the CRN are collaborating on grants, including one that has been funded by the European Union to develop patient-reported outcome measures for HI, raise awareness for glucose as a vital sign, and investigate a potential new photo-dynamic-therapy for HI (45).

Progress has been made in understanding the natural history of HI by expanding HIGR into 7 additional languages and increasing its functionality to collect continuous glucose monitoring and glucometer data streams.

In the area of universal newborn screening and efforts to prevent brain damage, a protocol has been developed to collect data to support a universal newborn screening approach for HI. In addition, data from ongoing studies related to newborn screening have been shared across multiple centers. Additionally, CRN members have signed on to letters calling for stricter hypoglycemia guidelines for birth centers to avoid preventable brain damage.

One key publication was the first International Guidelines for the Diagnosis and Management of Hyperinsulinism (4). The CRN is working with the authors to create a lay version and to ensure that the guidelines are disseminated worldwide. A consensus statement on global advocacy and access needs has been developed, which will be deployed to health ministries worldwide to ensure critical access to supplies, healthcare providers, and treatments. Both of these actions are in service of continually evolving global care guidelines and access to the necessary components of care.

Members of the CRN are also working together to develop guidelines for continuous glucose monitoring for HI and collect additional data on the current use of devices within the community. This effort will help capture the natural history of the disease and ensure better tools and strategies for glucose monitoring for patients and families.

Biotech members of the CRN are making progress in clinical research. One sponsor has completed Phase 3 clinical trials and has submitted a New Drug Application to the FDA. Another sponsor has multiple sites for a Phase 3 clinical trial. A third sponsor has multiple sites for a Phase 2 clinical trial. In all three cases, members of the CHI CRN are involved either as clinical research sites or as patient experience advisors.

Beyond current research and advocacy endeavors, the PRA process has laid the foundation for more long-term objectives and continually evolving technological and therapeutic improvements for those living with HI. The members of the CRN have been actively involved in launching new studies, growing their research programs, recruiting new researchers to the community, and making strides on other items within the PRA.

The community building facilitated by the establishment of the CRN and prioritized research agenda process helped CRN members identify areas of collaboration, increase trust to share data and in-progress research studies, and lay the groundwork for new avenues of discovery and advocacy. Through this active and multidimensional alliance, the CRN is re-imagining the future for people living with HI by improving outcomes through more timely and accurate diagnosis, more effective and less burdensome treatments, more easily obtainable expert care, and better tools to manage HI at home.

## List of congenital hyperinsulinism collaborative research network members

**CRN Core Team**

Diva D. De Leon-Crutchlow, MD, MSCE

Title: Chief of the Division of Endocrinology and Diabetes and Director of the Congenital Hyperinsulinism Center at Children’s Hospital of Philadelphia. Professor, Department of Pediatrics, Perelman School of Medicine at the University of Pennsylvania

Paul Thornton, MB, BCh, MRCPI

Title: Chief, Division of Endocrine and Diabetes, Medical Director of the Congenital Hyperinsulinism Center at Cook Children’s Medical Center

Julie Raskin, MA

Title: Chief Executive Officer at Congenital Hyperinsulinism International

Jennifer Schmitt

Title: Chief Operating Officer at Congenital Hyperinsulinism International

Tai Pasquini, MPA, PhD

Title: Chief Research Officer at Congenital Hyperinsulinism International

**Medical/Scientific Members**

Sir Al Aynsley-Green, MD

Title: Pediatric Endocrinologist; former President, British Medical Association; former Children’s Commissioner Consultant at Aynsley-Green Consulting

Jean Baptiste Arnoux, MD, MSc

Title: Pediatric Metabolic Hospital Practitioner at Necker University Hospital

Indraneel Banerjee, MBBS, MD, MRCPCH

Title: Pediatric Endocrinologist at Royal Manchester Children’s Hospital, Clinical Lead for the Northern Congenital Hyperinsulinism Service

Oliver Blankenstein, MD

Title: Pediatric Endocrinologist, Medical Director at Charité – Universitätsmedizin Berlin

Henrik Thybo Christesen, MD, PhD

Title: Consultant and Professor at Hans Christian Andersen Children’s Hospital, Odense University Hospital, Denmark

Louise S. Conwell, MBBS, PGCert MEd, FRACP, PhD

Title: Pediatric Endocrinologist, Queensland Children’s Hospital, Brisbane, Australia; Head, Children’s Health Queensland Clinical Unit, Faculty of Medicine, University of Queensland, Brisbane, Australia

Antonia Dastamani, MD, PhD

Title: Consultant in Paediatric Endocrinology and Diabetes, Lead of the Congenital Hyperinsulinism NHS Highly Specialised Service, Great Ormond Street Hospital for Children NHS Foundation Trust

Susann Empting, MD

Title: Pediatric Endocrinologist, Professor at Otto-von-Guericke University Magdeburg

Sarah E. Flanagan, PhD

Title: Professor of Genomic Medicine and Wellcome Trust Senior Research Fellow at the University of Exeter

Clare Gilbert, RN

Title: Clinical Nurse Specialist at Great Ormond St Hospital

David Gillis, MD

Title: Pediatric Endocrinologist at Hadassah Hebrew University Hospital, Associate Clinical Professor, Hebrew University

Theis Gondolf

Title: Senior Medical Director at Zealand Pharma

Deborah Harris, PhD FCNZ

Title: Neonatal Nurse Practitioner, Associate Professor Senior Lecturer, Deputy Director, Nursing Science Advanced Practice at School of Nursing, Midwifery and Health Practice, Victoria University of Wellington

Davelyn Hood, MD, MBA

Title: Director and Head of Scientific & Patient Affairs at Rezolute

Jelena Ivkovic, MD

Title: Senior Medical Director, Medical & Science at Zealand Pharma

Jeffrey R. Kaiser, MD, MA

Title: Professor of Pediatrics and Obstetrics and Gynecology, Chief of Neonatal-Perinatal Medicine, Penn State Health Children’s Hospital

Jennifer M. Kalish, MD, PhD

Title: Attending Physician at the Children’s Hospital of Philadelphia and Assistant Professor of Genetics and Pediatrics at the Perelman School of Medicine at the University of Pennsylvania

Nino Kheladze, MD

Title: Head of the Endocrinology and Diabetes Centre, M. Iashvili Children’s Central Hospital, Evex Medical Corporation

Mansa Krishnamurthy, MD, MSc

Title: Assistant Professor, Pediatric Endocrinology, Cincinnati Children’s Hospital Medical Center

Sebastian Kummer, MD

Title: Pediatric Endocrinologist at University Children’s Hospital Dusseldorf, Germany

Changhong Li, MD, PhD

Title: Associate Director at Nanjing Institute of Advanced Biotechnology and Life Science, China

Raphael Del Roio Liberatore Jr, MD, PhD

Title: Pediatric Endocrinologist at Ribeirão Preto Medical School- São Paulo University

Lauren N. Lopez, PhD

Title: Registry Director at Congenital Hyperinsulinism International

Katherine Lord, MD

Title: Pediatric Endocrinologist at Children’s Hospital of Philadelphia. Associate Professor, Department of Pediatrics, Perelman School of Medicine at the University of Pennsylvania

Seth Marks, MD, MSc

Title: Pediatric Endocrinologist at Children’s Hospital Health Sciences Centre Winnipeg. Associate Professor, Department of Pediatrics and Child Health, Rady Faculty of Health Sciences, University of Manitoba

Heather McKnight, MSN, CRNP

Title: Pediatric Nurse Practitioner at Children’s Hospital of Philadelphia

Thomas Meissner, MD

Title: Deputy Director Department of General Pediatrics, Neonatology and Pediatric Cardiology at University Children’s Hospital Düsseldorf

Klaus Mohnike, MD

Title: Pediatric Endocrinologist, Professor at Otto-von-Guericke University Magdeburg

Kate Morgan

Title: Clinical Nurse Specialist at Great Ormond St Hospital

Erin R. Okawa, MD

Title: Pediatric Endocrinologist, Director of the Hyperinsulinism Center at UCLA/Mattel Children’s Hospital

Jeff Roeseler

Title: Vice President of New Product Planning at Rezolute, Inc.

Kristen E. Rohli, PhD

Title: Research Manager at Congenital Hyperinsulinism International

Elizabeth Rosenfeld, MD, MSCE

Title: Pediatric Endocrinologist at Children’s Hospital of Philadelphia. Assistant Professor, Department of Pediatrics, Perelman School of Medicine at the University of Pennsylvania

Maria Salomon Estebanez, MD

Title: Pediatric Endocrinologist at Royal Manchester Children’s Hospital

Senthil Senniappan, MD, FRCPCH, MSc(Diab), PhD

Title: Pediatric Endocrinologist at Alder Hey Children’s Hospital

Pratik Shah, MB BS, DCH, MRCPCH, PhD

Title: Pediatric Endocrinologist at Barts Health NHS and Honorary Senior Lecturer at William Harvery Research

Show-Ling Shyng, PhD

Title: Professor of Chemical Physiology and Biochemistry at Oregon Health & Science University

Charles A. Stanley, MD

Title: Founder of the HI Center at Children’s Hospital of Philadelphia and Professor Emeritus of Pediatrics at the University of Pennsylvania Perelman School of Medicine

Lisa States, MD

Title: Pediatric Radiologist at Children’s Hospital of Philadelphia

Lisa Truong, CPNP-PC

Title: Nurse Practitioner at Cook Children’s Medical Center

Chris Worth

Title: Researcher and paediatric doctor at Royal Manchester Children’s Hospital

Daphne Yau, MD, MSc, FRCPC

Title: Pediatric Endocrinologist, Jim Pattison Children’s Hospital; Assistant Professor, University of Saskatchewan

David Zangen, MD

Title: Director of the division of Pediatric Endocrinology, Professor at Hadassah Hebrew University Medical center and Medical School

**Patient/Caregiver Members**

Lily Barnett

Title: Communications Associate at Congenital Hyperinsulinism International

Sheila Bose

Title: Caregiver Representative, CHI Board of Trustees, Secretary

Helen Couch, MB BChir MA (Cantab) MRCPCH

Title: HI Parent, Paediatric Registrar at Cambridge University Hospitals NHS Foundation Trust, UK

Sarah Dearman

Title: Chair of The Children’s Hyperinsulinism Charity

Michael Ferguson

Title: Caregiver Representative & Biopharma Strategist, Your Path Solutions

Leo Folsom, MPH

Title: Patient Representative

Carla Germanos

Title: Caregiver Representative

Ciara Grace

Title: Trustee of The Children’s Hyperinsulinism Charity

Janet Lee

Title: Caregiver Representative

Sandra Melo, PsyD

Title: Caregiver Representative

Cristina Pindado Gonzalez

Title: Caregiver Representative, President of AFHICO

Irene Promussas, PhD

Title: Caregiver Representative, Pharmacist, President of Lobby4kids

Ulrike Seyfarth

Title: Caregiver Representative, President of Kongenitaler Hyperinsulinismus e.V., Medical Technologists in Radiology (MTR) - Radiology/Nuclear Medicine at Charité – Universitätsmedizin Berlin

Lora (Bartocci) Van Arsdell, PsyD, LCSW

Title: Caregiver Representative

**Industry Members**

Hanmi

Rezolute

Zealand
